# Prevalence of Premenstrual Syndrome and Associated Factors Among Academics of a University in Midwest Brazil

**DOI:** 10.1055/s-0041-1741456

**Published:** 2022-02-25

**Authors:** Ana Paula Rodrigues Rezende, Fernanda Rassi Alvarenga, Marcelo Ramos, Débora Luiza Franken, Juvenal Soares Dias da Costa, Marcos Pascoal Pattussi, Vera Maria Vieira Paniz

**Affiliations:** 1 Faculdade de Medicina, Universidade de Rio Verde, Goianésia, GO, Brazil; 2 Faculdade de Medicina, Universidade de Rio Verde, Aparecida de Goiânia, GO, Brazil; 3 Programa de Pós-Graduação em Saúde Coletiva, Universidade do Vale do Rio dos Sinos, São Leopoldo, RS, Brazil

**Keywords:** premenstrual syndrome, premenstrual dysphoric disorder, cross-sectional studies, risk factors, students, síndrome pré-menstrual, transtorno disfórico pré-menstrual, estudos transversais, fatores de risco, estudantes

## Abstract

**Objective** To investigate the prevalence of premenstrual syndrome (PMS) and premenstrual dysphoric disorder (PMDD) in university students, the factors associated with PMS, the most prevalent symptoms, and the interference of symptoms in academic, family, social, and work activities.

**Methods** This cross-sectional study included 1,115 university students aged ≥ 18 years from the University of Rio Verde, Goiás. Premenstrual syndrome and PMDD were identified using the Premenstrual Symptoms Screening Tool. Associations with sociodemographic, behavioral, reproductive, nutritional, and health factors were investigated using the Poisson regression.

**Results** The prevalence of PMS was 46.9% (95% confidence interval [CI] 44.0–49.8), and of PMDD, 11.1% (95% CI 9.3–13.0). The most prevalent symptoms were physical, such as breast tenderness, bloating, e weight gain (73%); followed by psychological ones such as overeating/food cravings, tearful/more sensitive to rejection (> 60%). More than 30% of the patients reported that the symptoms interfered in a moderate-to-severe way in their social and academic activities. After adjusted analysis, PMS was more prevalent in those who were attending the 1 ^st^ /2 ^nd^ semester of college (prevalence ratio [PR] 1.44; 95% CI 1.14–1.80), those who consumed alcohol in the last 30 days (PR 1.23; 95% CI 1.04–1.47), and those who had depression (PR 1.49; 95% CI 1.30–1.71).

**Conclusion** Almost half of the university students had PMS and ∼ 11%, PMDD. Physical symptoms were the most common and interfered in a moderate-to-severe way in various aspects of life. Attending the first semesters, consuming alcohol, and having depression were risk factors for PMS. The identification of risk factors for PMS is essential to prevent symptoms and reduce the impact of the syndrome.

## Resumo

**Objetivo** Investigar a prevalência de síndrome pré-menstrual (SPM) e do transtorno disfórico pré-menstrual (TDPM) em alunas universitárias, os fatores associados à SPM, os sintomas mais prevalentes e a interferência dos sintomas nas atividades acadêmicas, familiares, sociais e de trabalho.

**Métodos** Este estudo transversal incluiu 1.115 estudantes universitárias ≥ 18 anos da Universidade de Rio Verde, Goiás. Síndrome pré-menstrual e TDPM foram identificados por meio do *Premenstrual Symptoms Screening Tool* . As associações com fatores sociodemográficos, comportamentais, reprodutivos, nutricionais e de saúde foram investigadas utilizando-se a regressão de Poisson.

**Resultados** A prevalência de SPM foi de 46,9% (intervalo de confiança [IC] de 95% 44,0–49,8) e de TDPM, 11,1% (IC 95% 9,3–13,0). Os sintomas mais prevalentes foram físicos, como sensibilidade mamária, distensão abdominal e ganho de peso (73%); seguidos por psicológicos, como comer demais/desejos por comida, chorar/mais sensível à rejeição (> 60%). Mais de 30% relataram que os sintomas interferiam de forma moderada a grave em suas atividades sociais e acadêmicas. Após análise ajustada, a SPM foi mais prevalente naquelas que estava cursando o 1°/2° semestre da faculdade (razão de prevalência [RP] 1,44; IC 95% 1,14–1,80), as que haviam consumido álcool nos últimos 30 dias (RP 1,23; IC 95% 1,04–1,47), e as que tinha depressão (RP 1,49; IC 95% 1,30–1,71).

**Conclusão** Quase metade das universitárias tinha SPM e cerca de 11%, TDPM. Os sintomas físicos foram os mais comuns e interferiram de forma moderada a grave em vários aspectos da vida. Frequentar os primeiros semestres, consumir álcool e ter depressão foram fatores de risco para SPM. A identificação dos fatores de risco para a SPM é essencial para prevenir os sintomas e reduzir o impacto da síndrome.

## Introduction

Premenstrual syndrome (PMS) is characterized by a set of physical and psychological symptoms, which start 1 to 2 weeks before menstruation, subside with the onset of menstrual flow, and are cyclical and recurrent. In addition to premenstrual tension, PMS can be severe enough to impact some aspects of a woman's life. 1

The etiology of PMS remains unknown, but the source of symptoms is associated with the susceptibility of some women to normal hormonal changes that occur during the menstrual cycle. Premenstrual symptoms often improve with the use of ovulation inhibitors during pregnancy and after menopause, which provides strong evidence of their relationship with cyclic ovarian function. In addition, the stabilization of neurotransmitters, such as serotonin, and changes in the effect of gamma-aminobutyric acid (GABA) through the use of antidepressant or anxiolytic drugs can improve the symptoms of PMS. Therefore, it appears that these neurotransmitters play an important role in the development of premenstrual symptoms. 2

The main symptoms observed in women with PMS are increased breast size and sensitivity, swelling, weight gain, headache, acne, anxiety, irritability, depression, mood swings, and changes in appetite. The intensity of symptoms varies among women. Many women have mild symptoms in their reproductive years, such as breast engorgement and edema, but do not perceive these symptoms as distressing or debilitating. However, other women may have intense and disabling symptoms, which may lead to the more severe form of PMS, that is, premenstrual dysphoric disorder (PMDD), 3 4 which is characterized by significant and debilitating psychiatric symptoms that can lead to disruptions in relationships, work, or social activities at levels similar to those of severe depression. 5

Confirming the diagnosis of PMS is important to differentiate it from other diseases, determine its prevalence, and define an effective treatment. 5 There are no definitive and universally accepted diagnostic criteria for PMS. Some criteria are comprehensive, based on the presence of only one symptom, and others are restrictive, taking into account the interference of symptoms in daily activities. 6 The existing criteria recognize a wide range of symptoms related to the syndrome, their severity, and their temporal pattern in parallel with the menstrual cycle, allowing the differentiation of clinically significant PMS from normal changes in the menstrual cycle. 6 7 Interviews, questionnaires, and self-assessment scales based on the criteria have been established for the diagnosis of PMS and PMDD. 8

In the available literature, variations in prevalence can be found. A review study found that the prevalence of physiological premenstrual symptoms varied between 50 and 85% , PMS varied between 30 and 40%, and PMDD varied between 3 and 8%. The absence of diagnostic consensus, differences in the interpretation of symptoms to define PMS, and the different populations investigated justify, at least in part, the inconsistencies in the prevalence of PMS between the studies of the review. 2

Some sociodemographic, reproductive, and behavioral characteristics are associated with the occurrence of PMS. Studies have shown that the prevalence of premenstrual symptoms is high in younger, black, and obese women; those with unhealthy eating habits; those who consume alcohol; and those who smoke. 3 9 10 11 12 13 On the contrary, the use of hormonal contraceptives and the practice of regular physical activities reduce the probability of occurrence of PMS. 14 15 Some studies have shown a direct association of socioeconomic level and education with PMS, while others showed an inverse association. 3 13 14

Regarding the impact of PMS symptoms, the literature shows that it can be severe enough to interfere with the relationships and daily activities of women of reproductive age. 16 17 These symptoms can also impair women's professional or academic performance and have economic consequences owing to increased absenteeism. 17

Therefore, the present study aimed to investigate the prevalence of PMS and PMDD in university students, the factors associated with PMS, the most prevalent symptoms, and the interference of symptoms in academic, family, social, and work activities.

## Methods

This was a cross-sectional university-based study performed with students of courses in the area of health from a university in the Midwest Brazil. The study is part of a larger project that evaluated the epidemiological profile of university students in the health field at Universidade de Rio Verde, Goiás, in 2018. The larger study included all university students (men and women) aged ≥ 18 years, from the courses of nursing, dentistry, medicine, physiotherapy, pharmacy, and physical education, from three campuses, who were attending the university from November 2018 and who agreed to participate in the research. The present study included female university students aged ≥ 18 years. University students who did not menstruate (due to health problems, use of continuous contraceptives, menopause, pregnancy, or breast-feeding) and those with irregular menstrual cycles (intervals shorter than 21 days or longer than 35 days, according to literature at the time of the study) were excluded because the instrument used to measure PMS has not been validated for this population. 3 11 18 19

To achieve the objectives of this study, the sample size was calculated adopting 40% prevalence for PMS and 16% for PMDD, with a margin of error of 3 percentage points, and a 95% confidence level, 20 resulting in a size of sample of 557 university students. The sample size was inflated by 25% to account for potential losses and refusals (10%) and to maintain statistical power after adjusting for confounding factors (15%). Thus, the sample size obtained allowed the detection of prevalence ratios 1.3 or higher, with a 95% confidence interval, with statistical power of 80%.

The fieldwork was performed by a trained field team, who approached the students on campuses. After receiving explanation of the nature of the study and having granted informed consent, the participants filled out a standardized, self-administered, and pretested questionnaire in a pilot study.

The study outcome was the prevalence of PMS and PMDD, measured by the Premenstrual Symptoms Screening Tool (PSST), which was validated for the Brazilian population. 18 19 The PSST consists of two domains: the first one comprises the 14 physical and psychological manifestations that reflect the Diagnostic and Statistical Manual of Mental Disorders, Fourth Edition (DSM-IV) criteria, while the 2 ^nd^ domain consists of 5 items that assess the functional impact of premenstrual symptoms. Each item is classified according to severity as “absent,” “mild,” “moderate,” or “severe.” The symptoms included were as follows: 1) anger/irritability, 2) anxiety/tension, 3) tearful/sensitive to rejection, 4) depressed/hopeless mood, 5) lack of interest in activities at work, 6) lack of interest in homework, 7) lack of interest in social activities, 8) difficulty concentrating, 9) fatigue/lack of energy, 10) overeating/food cravings, 11) insomnia, 12) hypersomnia (too much sleep), 13) feeling overwhelmed or out of control, and 14) physical symptoms (breast tenderness, headaches, joint/muscle pain, bloating, weight gain). The second domain included the impact of these symptoms on A) work/college efficiency or productivity, B) relationships with coworkers/colleagues, C) relationships with family, D) social life activities, and E) home responsibilities. In items A and B, the words “college” and “colleagues” were added to suit the reality of the population studied. To confirm the presence of PMS, the following criteria were established: the presence of 1) at least 5 symptoms (1–14), rated as moderate or severe; 2) at least 1 of the first 4 symptoms (1–4), rated as moderate or severe, and 3) at least 1 item from A to E, rated as moderate or severe. For PMDD, the following criteria were established: the presence of 1) at least 5 symptoms (1–14) rated as moderate or severe, 2) at least 1 of the first 4 symptoms (1–4) rated as severe, 3) and at least 1 item from A to E, rated as severe. 18 19

The sociodemographic characteristics assessed were age (18–20, 20.1–22.0, 22.1–24.0, or > 24 years), self-reported skin color (white or non-white), course period (1 ^st^ –2 ^nd^ , 3 ^rd^ –4 ^th^ , 5 ^th^ –6 ^th^ , 7 ^th^ –8 ^th^ , or ≥ 9 ^th^ ), and economic class (categorized using the Economic Classification Criterion of the Brazilian Association of Research Companies: A, B, C, D or E and recategorized later in A, B, C–E). 21 This classification criterion estimates the buying power of individuals and families including the possession of household items and the education level of the householder. The behavioral variables measured were smoking status (non-smoker: never smoked or smoked for less than a month; former smoker: stopped smoking; smoker: smokes for more than a month), alcohol consumption in the last 30 days (no: no cup or dose in the last 30 days/yes: at least one cup or dose in the last 30 days; one dose is equivalent to a can of beer or a glass of wine or a dose of cachaça or whiskey or others), physical activity (no: when they engaged in physical activities < 150 minutes per week, or yes: when they engaged in physical activities for ≥ 150 minutes per week), 22 and eating habits (unhealthy: consumption of vegetables and fruits < 5 times/week, or healthy: ≥ 5 times/week). 23 Reproductive variables included the use of hormonal contraceptives (do not use; use of one method; use of two or more methods, e.g., oral/injectable contraceptives/other methods, such as vaginal rings, implants, patches, and hormonal intrauterine device [IUD]), and age at menarche (≥ 12 years, or < 12 years). Nutritional status was assessed according to body mass index (BMI [kg/m ^2^ ]) and classified as normal weight (BMI < 25 kg/m ^2^ ), overweight (BMI ≥ 25 kg/m ^2^ and < 30 kg/m ^2^ ), and obesity (BMI ≥ 30 kg/m ^2^ ). 24 The health variables evaluated were medical diagnosis of hyperthyroidism or hypothyroidism (no/yes) and depression (no/yes), referred by the university students.

Data were compiled in the EpiData 3.1 software, by the double-data entry method, followed by comparison of entries and consistency analysis. The Stata 13.0 software (StataCorp LP, College Station, TX, USA) was used to describe the study population, the premenstrual symptoms investigated in the two PSST domains, and to calculate the prevalence of PMS and PMDD. The hierarchical approach 25 and Poisson regression 26 with variance were used for multivariate analysis. Socioeconomic variables were considered distal (level 1) determinants, behavioral and reproductive variables as intermediate (level 2) determinants, and nutritional and health variables as proximal (level 3) determinants. The effect of each variable on the sample was calculated by means of prevalence ratios and their respective 95% confidence intervals. All exposures (sociodemographic, behavioral, reproductive, nutritional, and health variables) under study were considered to be potential confounding factors. Variables with *p* < 0.20 on crude and adjusted analysis were carried forward to the multivariate model as potential confounders, and the significance level adopted was 5%.

This study was approved by the Research Ethics Committee at Universidade do Vale do Rio dos Sinos (UNISINOS), Protocol 2.892.764, which hosted the study's coordination, and Universidade de Rio Verde, Protocol 2.905.704, which hosted the study's data collection, in accordance with the National Health Council Resolution 466/12. All participants signed a consent form, guaranteeing confidentiality of information and the right to withdraw participation at any time.

## Results

In total, 1,596 university students were considered eligible. Of these, 481 met the exclusion criteria; thus, 1,115 university students were analyzed. The most prevalent characteristics found among university students were age range between 20.1 and 22 years (mean age 22.5 ± 3.4 years), white skin color, attending the 3 ^rd^ /4 ^th^ semester of college, and economic class B. Regarding behavioral characteristics, 62.7% were physically active, less than half had healthy eating habits (43.9%), more than 90% did not smoke, and 74% had consumed alcohol in the last 30 days. About two-thirds of the group used hormonal contraceptives, and more than 70% had menarche at age ≥ 12 years. Approximately 15% had BMI > 25 kg/m ^2^ , 7.9% had hypothyroidism or hyperthyroidism, and 20.6% were diagnosed with depression ( Table 1 ).

**Table 1:** Sociodemographic, behavioral, reproductive, nutritional, and health characteristics of students from a university in Midwest Brazil, 2018 ( *n* = 1,115)

Variables	n	%
Total	1,115	100
Age (years)		
18–20	212	19.0
20.1–22	386	34.6
22.1–24	294	26.4
> 24	223	20.0
Skin color		
White	653	58.6
Non-white	462	41.4
Course period (semester) ( *n* = 1,114 - uninformed: 1)		
≥ 9 ^th^	163	14.6
7 ^th^ and 8 ^th^	219	19.7
5 ^th^ and 6 ^th^	241	21.6
3 ^rd^ and 4 ^th^	262	23.5
1 ^st^ and 2 ^nd^	229	20.6
Economic class * ( *n* = 1,076 - uninformed: 39)		
A (high)	448	41.6
B	496	46.1
C–E (low)	132	12.3
Practice of physical activity ** ( *n* = 1,084 - uninformed: 31)		
No	404	37.3
Yes	680	62.7
Eating habits *** ( *n* = 1,114 - uninformed: 1)		
Unhealthy	625	56.1
Healthy	489	43.9
Smoking status ( *n* = 1,093 - uninformed: 22)		
Non-smoker	1,003	91.8
Former smoker	45	4.1
Smoker	45	4.1
Alcohol consumption (in the last month) ( *n* = 1,114 - uninformed: 1)		
No	290	26.0
Yes	824	74.0
Use of hormonal contraceptives ( *n* = 969 - uninformed: 146)		
Do not use	336	34.7
1	487	50.3
≥ 2	146	15.1
Menarche ( *n* = 1,106 - uninformed: 9)		
≥ 12 years	780	70.5
< 12 years	326	29.5
Nutritional status ( *n* = 1,095 - uninformed: 20)		
Normal	924	84.4
Overweight	135	12.3
Obesity	36	3.3
Hyperthyroidism/Hypothyroidism **** ( *n* = 1,098 - uninformed: 17)		
No	1,011	92.1
Yes	87	7.9
Depression **** ( *n* = 1,081 - uninformed: 34)		
No	858	79.4
Yes	223	20.6

* ABEP, Economic Classification Criterion of the Brazilian Association of Research Companies.

** Practicing some physical activity for at least 150 minute/week.

*** Healthy eating habits considered: consumption of fruits and vegetables ≥ 5 days/week.

**** Diagnosed by a doctor, as reported by the university student.

The prevalences of PMS and PMDD were 46.9% (95% CI, 44.0–49.8) and 11.1% (95% CI, 9.3–13.0), respectively, according to the PSST criteria. The physical symptoms (breast tenderness, headaches, joint/muscle pain, bloating, weight gain) were present in 73% of university students, and the most prevalent psychological symptoms were overeating/food cravings, tearfulness/sensitivity to rejection, anxiety/tension, and anger/irritability; in more than 60% of the cases in moderate or severe way. In more than 30%, these symptoms interfered with their efficiency and productivity at work/college, relationships with colleagues, family relationships, activities, and social life, in moderate-to-severe ways. Difficulty concentrating and feeling under pressure or severely out of control were reported by ∼ 8.5% and 8.4% of the students, respectively ( Table 2 ).

**Table 2:** Frequency of responses on premenstrual symptoms addressed in the domains of the premenstrual symptoms screening tool in students from a university in Midwest Brazil, 2018 ( *n* = 1,115)

	Absent n (%)	Mild n (%)	Moderate n (%)	Severe n (%)
**Premenstrual symptoms**				
Anger/irritability	120 (10.8)	304 (27.3)	492 (44.1)	199 (17.9)
Anxiety/tension	120 (10.8)	302 (27.1)	488 (43.8)	205 (18.4)
Tearful/more sensitive to rejection	114 (10.2)	235 (21.1)	471 (42.2)	295 (26.5)
Depressed mood/hopelessness	267 (24.0)	321 (28.8)	345 (30.9)	182 (16.3)
Lack of interest in activities at work	322 (28.9)	340 (30.5)	329 (29.5)	124 (11.1)
Lack of interest in homework	307 (27.5)	333 (29.9)	333 (29.9)	142 (12.7)
Lack of interest in social activities	313 (28.1)	368 (33.0)	312 (28.0)	122 (10.9)
Difficulty concentrating	418 (37.5)	322 (28.9)	278 (24.9)	97 (8.7)
Fatigue/lack of energy	271 (24.3)	331 (29.7)	381 (34.2)	132 (11.8)
Overeating/food cravings	90 (8.1)	239 (21.4)	438 (39.3)	348 (31.2)
Insomnia	584 (52.4)	274 (24.6)	193 (17.3)	64 (5.7)
Hypersomnia	422 (37.9)	291 (26.1)	268 (24.0)	134 (12.0)
Feeling overwhelmed	457 (41.0)	275 (24.7)	289 (25.9)	94 (8.4)
Physical symptoms: breast tenderness, headaches, joint/ muscle pain, bloating, weight gain.	86 (7.7)	220 (19.7)	439 (39.4)	370 (33.2)
**Interferes with**				
Work/college efficiency or productivity	278 (24.9)	438 (39.3)	346 (31.0)	53 (4.8)
Relationships with coworkers/colleagues	257 (23.1)	425 (38.1)	359 (32.2)	74 (6.6)
Relationships with family	233 (20.9)	422 (37.9)	376 (33.7)	84 (7.5)
Social life activities	248 (22.2)	450 (40.4)	349 (31.3)	68 (6.1)
Home responsibilities	366 (32.8)	442 (39.6)	250 (22.4)	57 (5.1)

The prevalence of PMS was higher in students who attended 1 ^st^ and 2 ^nd^ course periods (54.6%), had unhealthy eating habits (49.6%), were smokers (62.2%), and who consumed alcohol in the last month (49.4%). It was also prevalent in those who used one or more hormonal contraception methods (43.7% and 59.6%, respectively) and in those with depression (65%) ( Table 3 ).

**Table 3:** Prevalence and crude Poisson regression of premenstrual syndrome in relation to sociodemographic, behavioral, reproductive, nutritional, and health variables of students from a university in Midwest Brazil, 2018 ( *n* = 1,115)

Variables	Prevalence of PMS	PR (95% CI)	*p* -value
	**n (%)**		
Age (years)			0.325 a
18–20	102 (48.2)	1	
20.1–22	186 (48.2)	1.00 (0.84–1.19)	
22.1–24	137 (46.6)	0.97 (0.80–1.17)	
> 24	98 (44.0)	0.92 (0.74–1.12)	
Skin color			0.518 b
White	301 (46.1)	1	
Non-white	272 (48.1)	1.04 (0.92–1.18)	
Course period (semester)			0.001 a
≥ 9 ^th^	62 (38.0)	1	
7 ^th^ and 8 ^th^	99 (45.2)	1.19 (0.93–1.52)	
5 ^th^ and 6 ^th^	107 (44.4)	1.17 (0.92–1.49)	
3 ^rd^ and 4 ^th^	129 (49.2)	1.30 (1.03–1.63)	
1 ^st^ and 2 ^nd^	125 (54.6)	1.44 (1.14–1.80)	
Economic class			0.999 a
A (high)	207 (46.2)	1	
B	238 (48.0)	1.04 (0.91–1.19)	
C–E (low)	59 (44.7)	0.97 (0.78–1.20)	
Practice of physical activity *			0.869 b
No	191 (47.3)	1	
Yes	325 (47.8)	1.01 (0.93–1.41)	
Eating habits **			0.047 b
Unhealthy	310 (49.6)	1	
Healthy	213 (43.6)	0.88 (0.77–0.99)	
Smoking status			0.018 b
Non-smoker	458 (45.7)	1	
Former smoker	25 (55.6)	1.22 (0.93–1.59)	
Smoker	28 (62.2)	1.36 (1.07–1.73)	
Alcohol consumption (in the last month)			0.008 b
No	116 (40.0)	1	
Yes	407 (49.4)	1.23 (1.06–1.44)	
Use of hormonal contraceptives			0.036 a
Do not use	153 (45.8)	1	
1	213 (43.7)	0.95 (0.82–1.11)	
≥ 2	87 (59.6)	1.30 (1.09–1.55)	
Menarche			0.064 b
≥ 12 years	353 (45.3)	1	
< 12 years	167 (51.2)	1.13 (0.99–1.29)	
Nutritional status			0.062 a
Normal	422 (45.7)	1	
Overweight	75 (55.6)	1.22 (1.03–1.44)	
Obesity	18 (50.0)	1.09 (0.78–1.53)	
Hyperthyroidism/Hypothyroidism ***			0.206 b
No	467 (46.2)	1	
Yes	46 (52.9)	1.14 (0.93–1.41)	
Depression ^****^			< 0.001 b
No	354 (41.3)	1	
Yes	145 (65.0)	1.58 (1.39–1.79)	

Abbreviations: 95% CI, 95% confidence interval; PR, prevalence ratio.

* Practiced some physical activity for at least 150 minute/week.

** Healthy eating habits considered: consumption of fruits and vegetables ≥ 5 days/week.

*** Diagnosed by a doctor, as reported by the university student.

a Wald test, *p* -value for linear trend.

b Wald test, *p* -value for heterogeneity.

After adjusting for confounding factors, being in the first periods of the academic course (PR 1.44, 95% CI 1.14–1.80), alcohol consumption (PR 1.23, 95% CI 1.04–1.47), and medical diagnosis of depression (PR 1.49, 95% CI 1.30–1.71) remained associated with PMS. ( Table 4 ). The use of hormonal contraceptives and PMS did not maintain a direct linear association after adjustment. However, women who reported using 2 or more hormonal contraceptives were 22% more likely to have PMS compared with those who reported not using hormonal contraceptives (PR 1.22; 95% CI 1.02–1.46).

**Table 4:** Adjusted Poisson regression for premenstrual syndrome in relation to sociodemographic, behavioral, reproductive, and health variables of students at a university in Midwest Brazil, 2018 ( *n* = 1,115)

Level	Variables	PR (95% CI)	*p* -value
1	Course period (semester)		0.001 a
	≥ 9 ^th^	1	
	7 ^th^ and 8 ^th^	1.19 (0.93–1.52)	
	5 ^th^ and 6 ^th^	1.17 (0.92–1.49)	
	3 ^rd^ and 4 ^th^	1.30 (1.03–1.63)	
	1 ^st^ and 2 ^nd^	1.44 (1.14–1.80)	
2	Eating habits *		0.058 b
	Unhealthy	1	
	Healthy	0.88 (0.76–1.00)	
	Alcohol consumption (last 30 days)		0.018 b
	No	1	
	Yes	1.23 (1.04–1.47)	
	Use of hormonal contraceptives		0.115 a
	No		
	1	0.94 (0.80–1.10)	
	2 or +	1.22 (1.02–1.46)	
	Menarche		0.094 b
	≥ 12 years	1	
	< 12 years	1.13 (0.98–1.30)	
3	Depression **		< 0.001 b
	No	1	
	Yes	1.49 (1.30–1.71)	

Abbreviations: 95% CI: 95% confidence interval; PR, prevalence ratio.

Each variable was adjusted to the others at the same or previous level in a hierarchical model of causality. Only variables associated with the outcome at *p* < 0.20 in the unadjusted model were subsequently entered and retained in the final multivariate-adjusted model.

* Healthy eating habits considered: consumption of fruits and vegetables ≥ 5 days/week.

** Diagnosed by a doctor, as reported by the student.

a Wald test, *p* -value for linear trend.

b Wald test, *p* -value for heterogeneity.

## Discussion

The present study found a high and consistent prevalence of PMS among university students in the health field. The main complaints reported were physical (sensitive breasts, headache, muscle, or joint pain, swelling, and weight gain) and psychological symptoms (overeating/food cravings, tearfulness/sensitivity to rejection, anxiety/tension, and anger/irritability). These symptoms interfered in moderate-to-severe ways in the academic, family, and social activities of this population. After adjustment, the highest PMS probability occurred for university students who were attending the first periods of the course, who had consumed alcoholic beverages in the last 30 days, and who were diagnosed with depression.

The prevalence of PMS (46.9%) identified in the present study is consistent with that in the literature. One systematic review identified a combined prevalence of PMS of 47.8% (95% CI 32.6–62.9,), 20 which was similar to the value found in our study. In this review of 17 articles, the lowest prevalence reported was in France, at 12% (95% CI 11–13), 27 and the highest in Iran, at 98% (95% CI 97–100). 28 The reviewed studies used different methodologies, populations, assessment tools, and outcome categorization instruments, which may explain the wide difference in the prevalence rates. The prevalence of PMDD (11.1%) was lower than that reported in a study conducted in the Northeast of Brazil, with health professionals and university students (16.5%). 19

In relation to symptoms, the most prevalent ones found in this study were physical symptoms (tender breasts, headache, muscle, or joint pain, swelling, and weight gain), followed by desire for overeating/food cravings, tearfulness/sensitivity to rejection, anxiety/tension, and anger/irritability, which is consistent with the literature. A prospective, observational study involving 60 university students from Centro Universitário São Camilo, in São Paulo, with a mean age of 24.6 ± 4.7 years, identified the following main symptoms of PMS: irritability (76.6%), swelling (65%), and anxiety (58.3%). 4 A cross-sectional population study performed in Pelotas, RS, with 1,395 women aged between 15 and 49 years found the following main premenstrual symptoms: irritability, abdominal discomfort, nervousness, headache, tiredness, and mastalgia, all with a prevalence > 50%, 3 data similar to that found in this study.

Upon analyzing the associated factors, a high prevalence of PMS was found among university students in the initial periods of the course compared with those attending the final semesters. The association between lower educational levels and PMS has been described previously. In a population study performed in the United Kingdom, an inverse linear association was found in which the lowest level of education was associated with a high occurrence of PMS. 14 However, a Brazilian study identified a high prevalence of PMS in women with higher education. 2 It is worth mentioning that in the present study, the schooling indicator evaluated was the period of the course, since the population was composed only of university students. We found that university students from the first periods (1st and 2nd semesters) are susceptible to premenstrual symptoms in the same way that they are to depression and anxiety disorders. The higher probability of depressive symptoms and anxiety in freshmen than in university students in the last semesters has been reported in previous study. 29 Sex steroids and their receptors are abundant in the brain; they regulate emotions and behaviors and modulate the secretion of serotonin, which is implicated in the etiology of PMS. 30 As the first semesters are adaptation periods, these university students could be susceptible to these changes.

In relation to behavioral variables, an association was also found between alcohol consumption and PMS, which has already been evidenced. In a systematic review, 19 studies were evaluated, and it was found that alcohol consumption was associated with a 50% increase in the likelihood of developing PMS (odds ratio [OR] = 1.45; 95% CI 1.17–1.79). 12 Alcohol consumption can change the levels of sex steroid hormones and gonadotropin during the menstrual cycle, which may increase the risk of PMS occurrence, since the etiology of this syndrome is linked to fluctuations of these sex hormones during the menstrual cycle. 31 In addition, alcohol intake may increase the risk of PMS through its effect on serotonin and GABA activity, since these neurotransmitters are involved in the etiology of PMS. 12

Regarding the morbidities assessed, depression was associated with PMS after adjusting for possible confounding factors. This finding is consistent with another study that shows that university students with depressive symptoms are more likely to have symptoms of PMS and its most severe form, PMDD, which has already been classified as a depressive disorder. 32 In addition, it has already been shown that women with PMS have a higher risk of depression than those without PMS. 33 Although there is a clear association between depressive disorders and PMS in the literature, it is not yet clear whether these conditions predispose one to PMS or whether PMS increases the likelihood of these disorders. 30 Longitudinal studies are necessary to understand this relationship.

Contrary to what would be expected in relation to the use of hormonal contraceptive, there was no dose-response effect between the categories of this variable and PMS, through adequate statistical test. However, it was observed that women who reported using 2 or more hormonal contraceptives showed 22% more probability of having PMS than those who did not, even after adjustment for confounding factors. As there is no knowledge of a medical recommendation for the use of an overlap of hormonal contraceptive methods, a possible explanation for the association found for this category would be that women who present premenstrual symptoms replace hormonal methods more frequently during life, in search of a pharmacotherapy that fulfills the contraceptive function and reduces the premenstrual symptoms. Thus, it cannot be said that the use of a combination of hormonal contraceptives occurred, but that the woman experienced the use of more than one hormonal method.

In the present study, no association was found between smoking, eating habits, physical activity, and PMS, which was also demonstrated by other authors. 14 34 University students with healthy eating habits were less likely to have PMS, but after adjustment, this association showed a borderline *p* -value. Smoking was associated with PMS only in the crude analysis, probably because of the low prevalence of university smokers (4%), which gave little power to this analysis.

One of the main limitations of this study was its cross-sectional design since the association between PMS and some associated factors, such as depression, may have been affected by reverse causality as exposure and outcome were measured at the same time. Another limitation was the use of an instrument with retrospective information. There is evidence that data collected prospectively show a prevalence different from that of data obtained retrospectively. 8 However, PSST has been used in other studies and is considered a quick and easy-to-use assessment tool, which is valid for the screening of PMS/PMDD in Brazilian women. 18 19 Furthermore, all university students who did not menstruate but could still report the presence of premenstrual symptoms were excluded. However, this strategy sought to avoid memory bias inherent to studies that use recall. Finally, university students with a medical diagnosis of depression were not excluded. The exclusion of depressed subjects is indicated when using this type of instrument to distinguish the underlying psychiatric disorders that could be exacerbated in the premenstrual period. 32 However, the non-exclusion in our study allowed us to determine the independent association between depression and PMS in university students.

This study has several strengths. First, this is one of the few Brazilian studies that, in addition to determining the prevalence of PMS and PMDD in a population of young university students, evaluated the association of PMS with sociodemographic, reproductive, behavioral, nutritional, and health variables. Thus, this study expands the knowledge on PMS and the associated factors in Brazilian university students. Second, the significant sample size added power to the investigated associations. Third, the rigorous application of the methodology, using a validated instrument for the diagnosis of PMS, and controlling possible confounding factors, made it possible to analyze the independent effects of the variables.

## Conclusion

In conclusion, the study identified that almost half of the university students had PMS, and ∼ 11% had PMDD. Physical symptoms were more prevalent than psychological ones. These symptoms interfered in a moderate-to-severe manner in their social and academic activities. The university students who were at the beginning of the course, who consumed alcohol as well as those who had a diagnosis of depression had a higher prevalence of premenstrual symptoms; therefore, this population should be better monitored. The identification of risk factors for PMS is essential to prevent symptoms and reduce the impact of the syndrome. However, longitudinal studies are necessary to better elucidate the associations described here.
